# Underutilization of diagnostic assays for celiac disease in Korea

**DOI:** 10.1002/jcla.23913

**Published:** 2021-10-24

**Authors:** Rihwa Choi, Sang Gon Lee, Eun Hee Lee

**Affiliations:** ^1^ Department of Laboratory Medicine and Genetics Samsung Medical Center Sungkyunkwan University School of Medicine Seoul Korea; ^2^ Department of Laboratory Medicine Green Cross Laboratories Yongin Korea; ^3^ Green Cross Laboratories Yongin Korea

**Keywords:** celiac disease, endomysial antibody, gliadin antibody, test utilization, transglutaminase antibody

## Abstract

**Background:**

Test utilization for the diagnosis of celiac disease may affect the prevalence and incidence of the disease in Korea. We aimed to investigate the test utilization of serological biomarkers for celiac disease in Korea.

**Methods:**

We retrospectively investigated the test utilization of tissue transglutaminase IgA, gliadin IgA and IgG, and endomysial IgA antibody (Ab) assays between January 2011 and June 2020.

**Results:**

During a nine‐year‐and‐six‐month study period, overall 307,322,606 clinical tests were requested from different clinical settings, such as local clinics, hospitals, university hospitals, and tertiary medical centers. Among them, only 58 tissue transglutaminase IgA, 22 gliadin IgA, 12 gliadin IgG, and 16 endomysial IgA Ab tests were performed on 79 Korean patients. Among them, one patient had positive transglutaminase IgA Ab result (1.3%).

**Conclusion:**

Low prevalence and incidence of celiac disease in Korea may be due to an underutilization of diagnostic assays.

## Graphical abstract

Test utilization for the diagnosis of celiac disease may affect the prevalence and incidence of the disease in Korea. During a nine year and six month study period, overall 307,322,606 clinical tests were requested from different clinicalsettings, such as local clinics, hospitals, university hospitals, and tertiary medical centers. Among them, only 79 Korean patients were tested for celiac disease using tissue transglutaminase IgA, deaminatedgliadin IgA and IgG, and endomysial IgA antibody assays, revealing that these tests were extremely underutilized inKorea.

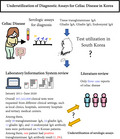

## 1 INTRODUCTION

Celiac disease is an autoimmune disorder involving the multisystem of the body, which is triggered by an immune reaction to a gluten‐containing diet occurring in genetically predisposed individuals (HLA‐DQ2/DQ8).^1^, ^2^ Clinical gastrointestinal manifestations of celiac disease, such as chronic diarrhea, malnutrition, and abdominal distention, are usually non‐specific symptoms.^3^, ^4^ For the diagnosis of celiac disease in adults, a combination of celiac disease serology testing and duodenal biopsy sampling is performed.^1^ According to a recent clinical practice guideline on diagnosis and monitoring of celiac disease by the American Gastroenterological Association, serology testing such as that of tissue transglutaminase IgA, deaminated gliadin IgA, deaminated gliadin IgG, and endomysial IgA antibodies (Abs) is an essential component of the detection and diagnosis of celiac disease.^4^ It has been reported that the prevalence of celiac disease is about 1.0% of the general population globally.^1^, ^4^ Meanwhile, in Asian countries, it has been reported that celiac disease is extremely uncommon in Asian populations; however, with the aid of serological biomarkers such as tissue transglutaminase IgA, deaminated gliadin IgA, deaminated gliadin IgG, and endomysial IgA Ab, it has been shown that celiac disease is both a common and an often underdiagnosed disorder in Asia.^3^, ^5^, ^6^, ^7^

In South Korea, there have been few case reports of celiac disease patients.^8^, ^9^, ^10^ However, to the best of our knowledge, there has been no information regarding the test utilization of diagnostic assays for celiac disease in Korea. Therefore, for the first time, in this study we aimed to investigate the test utilization of serological biomarkers including tissue transglutaminase IgA, deaminated gliadin IgA and IgG, and endomysial IgA Ab assays in South Korea.

## 2 MATERIALS AND METHODS

We retrospectively reviewed data for tissue transglutaminase IgA, deaminated gliadin IgA and IgG, and endomysial IgA Ab assays between January 1, 2011, and June 30, 2020, through Green Cross Laboratories’ information system. Green Cross Laboratories, one of the largest referral clinical laboratories in South Korea, provides clinical specimen analytical services for various types of assays including esoteric laboratory tests nation‐wide. All data were anonymized prior to being adopted for statistical analysis. The numbers and percentages of each considered test were presented in this study. The protocol of this study was approved by the institutional review board (IRB) of Green Cross Laboratories (GCL‐2020‐1043). A waiver of informed consent was approved by the IRB since the use of a waiver would not adversely affect the rights or welfare of the study subjects because the study was retrospective and involved minimal risk to the subjects.

## 3 RESULTS

During a nine‐year‐and‐six‐month study period, overall 307,322,606 clinical tests were requested from different clinical settings, such as local clinics, hospitals, university hospitals, and tertiary medical centers. Among them, only 90 tissue transglutaminase IgA, 25 deaminated gliadin IgA, 15 deaminated gliadin IgG, and 64 endomysial IgA Ab tests from 152 adult patients (79 Korean and 73 foreign patients) were requested. Among 79 Koreans, two patients obtained results for all four serologic assays, two patients had tissue transglutaminase IgA, deaminated gliadin IgA, and endomysial IgA Ab results, three patients had tissue transglutaminase IgA, deaminated gliadin IgA and IgG Ab results, and six patients had tissue transglutaminase IgA and endomysial IgA Ab results. Only 58 transglutaminase IgA, 22 deaminated gliadin IgA, 12 deaminated gliadin IgG, and 16 endomysial IgA Ab tests were performed in 79 Korean patients. Among all 79 Korean patients, no patients had positive results for any of the four assays except for one (1.3%) woman who had a positive tissue transglutaminase IgA Ab (4 U/ml, reference for negative result <4 U/ml). During the study period, all four assay tests were referred to Quest Diagnostics in the United States because clinical requests for these tests rarely occur in Korea and no clinical laboratory in South Korea provided analytical services for these tests.

## 4 DISCUSSION

Considering the annual number of clinical sample requests to Green Cross Laboratories, test utilization of these assays was extremely rare (<0.0001% for all clinical tests during the study period). This underutilization may be one of the main causes for the low disease prevalence and incidence of celiac disease in Korea.

In this study, one patient had a positive tissue transglutaminase IgA Ab result, though it showed a low titer value (<2 times the upper limit of normal).^11^ This result needed to be confirmed as celiac disease using intestinal biopsy.^4^ Unfortunately, clinical information is limited for further diagnostic approaches for celiac disease including biopsy in this patient.

It has been reported that the prevalence and incidence of celiac disease in Korea is extremely rare (Table 1), with the hypothesis that the prevalence of at‐risk patients with a genetic predisposition (HLA‐DQ2/DQ8) is low and a relatively low intake of foods containing gluten, which may lead physicians to overlook celiac disease during routine clinical practice and medical training.^6^, ^7^, ^8^, ^9^, ^10^, ^11^, ^12^ However, another important factor for the prevalence and incidence of celiac disease in Korea is the applicability of serologic assays to discover patients. In Korea, assays performed in overseas laboratories are not reimbursed by the single government payer and require out‐of‐pocket payment.^13^ Due to underutilization, clinical laboratories in Korea could not perform these serologic tests and have referred them to overseas laboratories. This has led to an increase in test costs for shipping and delayed turnaround times to report the results of the assays. The cost for each single serologic assay to diagnose celiac disease using overseas testing is higher than that of a duodenal biopsy in Korea. High costs and delayed turnaround times may lead to an underutilization of assays to diagnose celiac disease patients, resulting in insufficient awareness of the disease during training and clinical practice.

**TABLE 1:** Review of the literature on cases of celiac disease in Korea

Reference	Age	Sex	Serologic assay tested	Serologic assay results	Other modalities for the diagnosis of celiac disease
Gweon et al., 2013^8^	36	F	Endomysial IgA antibody Tissue transglutaminase IgA antibody	2 RU/ml (reference <20 RU/ml) Negative	D‐xylose absorption test, intestinal biopsy, and symptom resolution after gluten‐free diet
Hwang et al., 2015^9^	47	F	Endomysial IgA antibody Tissue transglutaminase IgA antibody	Negative Negative	HLA‐DQ2.5 genotype, intestinal biopsy, andsymptom resolution after gluten‐free diet
Ham et al., 2017^10^	60’s^a^	M	Endomysial IgA antibody	1:160 (reference <1:40)	Intestinal biopsy and symptom resolution after gluten‐free diet

^a^ Exact information about age was not available.

The results of this study offer basic information regarding the utilization of assays in association with celiac disease to improve understanding with regard to the prevalence and incidence of celiac disease based on test utilization of patients with celiac disease in Korea.

## CONFLICT OF INTEREST

None declared.

## AUTHOR CONTRIBUTIONS

All authors contributed to manuscript preparation; R. Choi, S.G. Lee, and E. H. Lee collected the data or contributed to data analysis; R. Choi and S.G. Lee designed the study; R. Choi, S.G. Lee, and E. H. Lee had full access to all the data in the study and take responsibility for the integrity of the data and the accuracy of the data analysis. All authors read and approved the final manuscript.

## Data Availability

The datasets generated and analyzed during the current study are available from the corresponding authors on reasonable request.
